# Establishment of hematological reference intervals for healthy adults in Asmara

**DOI:** 10.1186/s13104-018-3142-y

**Published:** 2018-01-22

**Authors:** Nejat Siraj, John Issac, Mohammed Anwar, Yohannes Mehari, Shushan Russom, Semere Kahsay, Haben Frezghi

**Affiliations:** Department of Clinical Laboratory Sciences, School of Allied Health Professions, Asmara College of Health Sciences (ACHS), P.O. Box 8566, Asmara, Eritrea

**Keywords:** Reference intervals, Asmara, Out of range, Red blood cell count, Hemoglobin

## Abstract

**Objectives:**

Clinical laboratory reference intervals used in a specific area should be derived from the local population as they are influenced by many factors. The purpose of this quantitative cross sectional study was to establish hematological reference intervals for healthy adults in Asmara and to determine whether the currently used reference interval do represent the adult population in the city. In addition, the established reference intervals were compared to findings from similar studies conducted in selected countries in Africa.

**Results:**

There was a significant difference between males and females in the reference intervals for erythrocyte count, hemoglobin, hematocrit, mean cell volume, mean cell hemoglobin, mean cell hemoglobin concentration and differential white blood cell count. All the evaluated hematological analytes were found to be higher in males than in females except for platelet count. The out of range percentage for the parameters extends from 3.5 to 46.7%; with red blood cell count having the lowest while mean cell volume having the highest out of range percentage. The results indicated that the currently used reference interval does not represent the population in Asmara and are different from those obtained elsewhere in Africa.

**Electronic supplementary material:**

The online version of this article (10.1186/s13104-018-3142-y) contains supplementary material, which is available to authorized users.

## Introduction

Clinical laboratory reference intervals (RI) play a great role in patient diagnosis, management, disease prognosis, monitoring of response to therapy and in monitoring possible adverse reactions to therapy [1]. However, hematological RI can be influenced by a variety of factors. Several investigators have reported an association between ethnic background, gender and environmental factors (altitude) and specific hematological indices. Others have concluded that genetic factors contribute to all blood cell measure differences, and may account for between 61 and 96% of the observed variance [2, 3]. Cognisant of this fact, the Clinical and Laboratory Standards Institute (CLSI) recommends that each laboratory should establish its own RI [4].

However, in most African countries, the RI for healthy populations used in most clinics rely upon values generated from Caucasian populations living in Western Europe of North America [5]. Admittedly, a limited number of studies have been conducted in the last decade in sub-Saharan Africa (SSA) [1, 5–8]. These investigators have demonstrated the existence of significant inter-country/regional variation in multiple haematological parameters for infants, children and adult populations.

In general, these studied have amplified the existing concern that utility of haematological RI in clinical settings across SSA may be compromised by the widespread use of non-population appropriate haematological RI. In addition, several investigators have opined that population-appropriate RI may have utility in the increasing number of clinical trials being conducted in populations domiciled in SSA [1, 5]. This suggestion is premised on the fact that toxicity tables used in adverse event reporting for these studies are based on RI derived from populations in Western countries [1].

The situation highlighted above applies broadly to Eritrea. In particular, laboratories in Asmara use hematological RI obtained either from textbooks or from reagent kit inserts. Therefore, the need to evaluate the applicability of the RI to the local population is clearly warranted. A need this paper was designed to meet. To our knowledge, this is the first study to establish haematological RI for adult populations in Eritrea. A comparison between the values obtained in this study and studies from selected African countries was also attempted.

## Main text

### Materials and methods

#### Study design and study population

This was a quantitative cross-sectional study and was conducted from March to May 2015 in Asmara, the capital city of Eritrea. The city is located at an altitude of 2230 m above sea level, on the edge of the Great Rift Valley.

Participants in this study included voluntary college students, laboratory staff members, ACHS staff members, factory workers and school teachers of both sexes with ages ranging between 18 and 49 years.

Potential participants who consented to the study were invited to the administrative center, where they were evaluated. A total of 600 volunteers who were residents in the area for at least 6 months were recruited in the study (Additional file 1). The proposed number was above the CLSI guidelines for the establishment of RI [4] which recommends a minimum of 120 participants in each category used for stratification.

All the participants met a pre-set clinical history; physical examination and CLSI inclusion and exclusion criteria. The exclusion criteria included: the presence of any disease including: anemia, cardio-vascular disease and high blood pressure. Additional exclusion criteria included: drug therapy, alcohol consumption; heavy smokers, chronic diseases such as diabetic mellitus (DM); individuals who had donated blood in the last 3 months. Individuals who had undergone surgery in the recent past were also excluded.

#### Blood collection and laboratory analysis

Four millilitres of venous blood was collected a Vacutainer system (Becton–Dickinson Biosciences, San Jose, CA, USA) in Dipotassium Ethylene Diaminetetraacetic acid (EDTA) tubes. The samples were collected between (9:00 a.m. and 11:30 a.m.). After mixing the blood with the anticoagulant; the test tubes were appropriately labeled. The samples were then placed in ice-box, transported to laboratory and processed within 2 h.

Haematological analysis was performed using (Beckman Coulter: AU 480 Chemistry System) at the Biet Mekae Community Hospital laboratory. The analyzed hematological parameters included: red blood cell (RBC) count, hemoglobin (Hgb), hematocrit (Hct), mean corpuscular volume (MCV), mean corpuscular hemoglobin (MCH), mean corpuscular hemoglobin concentration (MCHC), red cell distribution width (RDW), total white blood cell (WBC) count, differential WBC count (lymphocytes, granulocytes, monocytes) and platelet count.

Serological tests for hepatitis B virus (HBV) and hepatitis C virus (HCV) were undertaken using Determine™ HBsAg (Abbott Laboratories) and OraQuick^®^ HCV tests respectively.

#### Quality control

The analyzer was calibrated each day using commercial standards recommended by the manufacturer. Normal, abnormal low and abnormal high controls were run daily to monitor the accuracy of the analyzer. As part of external quality control, 10% of the sample collected was re-analyzed in Blood Bank quality control laboratory using the same type of hematological analyzer.

#### Data management and statistical analysis

Laboratory results were statistically analyzed using SPSS Version 20 program (SPSS version 20.0, SPSS Inc. Chicago, IL, USA). The analysis was informed entirely by CLSI (C28-A3) guidelines which recommend the use of 2.5th and 97.5th‰ and associated group comparisons based either on parametric or non-parametric statistics depending on whether the distribution of the data is Gaussian or non-Gaussian [4]. Normality plots, Kolmogorov–Smirnov and Shapiro–Wilks test were used to evaluate data distribution. Dixon method was used to identify outliers. The mean, median, standard deviation (SD), range, 2.5th and 97.5th‰ were subsequently evaluated. Differences between males and females were evaluated using the Mann–Whitney U test or student *t* test depending on data distribution. A two-sided p-value of < 0.05 was considered significant. The proportion of Out of Range (OOR) values was then calculated. The study consensus RI were subsequently compared to values currently in use by the Eritrean ministry of health—based on Beckman Coulter: AU 480 Chemistry System reagents inserts), specific African countries.

## Results

A total of 600 participants among the age group of 18–49 years participated in the study. The final sample size came out to be 591 as 9 outliers were excluded. Total 296 females and 295 males with the mean age of 33.4 ± 9.21 years were included. Stratified according to occupation, the study included: 216 college students (108 males and 108 females); 119 laboratory staff members (60 males and 59 females); 35 staff members of ACHS (16 males 10 and females); 233 factory workers and school teachers (111 males and 110 females). All the participants were sero-negative for HBV and HCV.

The 95th‰ interval for males in RBC count, Hgb, Hct, MCH, MCHC, differential lymphocyte are shown in Tables 1 and 2. There was a significant difference in the RBC count (p < 0.000); Hemoglobin (p < 0.003); Hct (p < 0.000); MCH (p < 0.005), MCHC (p < 0.001), and differential Lymphocyte count (p < 0.008) and granulocyte count (p > 0.024) between the two sexes. There was no significant difference between male and females in WBC (p > 0.838), MO (p > 0.903), platelet (p > 0.092), RDW (p > 0.775) and MCV (p > 0.650).

**Table 1:** Calculated hematological (RBC and RBC indices) RI for the screened population

Parameter	Sex	N	Mean	95% CI for mean	Median	Range	2.5th–97.5th‰	p-value (gender)
Red blood cells (RBC) (× 10^12^/L)	Combined	591	4.9	4.9–5.04	5.04	3.4–6.4	4.07–6.02	0.000
	Male	295	5.3	5.2–5.3	5.3	4.02–6.4	4.2–6.07	
	Female	296	4.7	4.7–4.8	4.7	3.2–6.1	4–5.7	
Hemoglobin (Hgb) (g/dL)	Combined	591	15.2	15.05–15.3	15.4	9.1–19.1	12.6–17.7	0.003
	Male	295	15.4	15.2–15.5	15.7	9.9–19.1	12.6–17.8	
	Female	296	14.9	14.8–15.2	15	9.1–18.4	12.5–17.6	
Hematocrit (Hct) (%)	Combined	591	46.7	46.4–47.1	47.3	31.1–59.3	38.3–54.4	0.000
	Male	295	49.3	48.8–49.7	49.9	38.4–59.3	40.5–55	
	Female	296	44.2	43.8–44.7	44.5	31.1–55.7	37.9–52	
Mean cell volume (MCV) (fL)	Combined	591	93.7	93.4–93.8	93.7	82.2–100.8	85.8–100	0.650
	Male	295	93.8	93.3–94.2	93.6	83.6–100.2	85.7–100	
	Female	296	93.6	93.2–94.0	93.8	82.2–100.8	85.5–100	
Mean cell hemoglobin (MCH) (g/dL)	Combined	591	30.6	30.5–30.7	30.6	26.1–33.5	27.4 –32.8	0.005
	Male	295	30.8	30.6–30.9	30.8	26.3–33.5	28–33	
	Female	296	30.4	30.2–30.5	30.5	26.1–33.5	26.5–32.6	
Mean cell hemoglobin concentration (MCHC) (g/dL)	Combined	591	32.6	32.6–32.7	32.9	29.5–34.3	30.2–33.8	0.001
	Male	295	32.8	32.7–32.9	33.0	29.6–34.3	30.4–33.7	
	Female	296	32.4	32.3–32.6	32.8	29.5–34.1	30–33.7	
Red cell distribution width (RDW) (%)	Combined	591	13.6	13.5–13.7	13.5	11.6–19.1	12.3–15.6	0.775
	Male	295	13.6	13.5–13.7	13.5	11.8–16.2	12.3–15.5	
	Female	296	13.6	13.5–13.7	13.4	11.6–19.1	12.3–17

**Table 2:** Calculated hematological (WBC and platelet) RI for the screened population

Parameter	Sex	N	Mean	95% CI for mean	Median	Range	2.5th–97.5th‰	p-value (gender)
White blood cells (WBC) (× 10^9^/L)	Combined	591	5.6	5.6–6.0	5.6	2.6–10.5	3.4–9.0	0.838
	Male	295	5.9	5.7–6.06	5.6	3–12.7	3.7–9.3	
	Female	296	5.8	5.6–6.0	5.6	2.6–10.1	3.3–8.9	
Lymhocyte (LY) (%)	Combined	591	39.2	38.4–39.0	37.3	17.3–66	22–59.2	*0.008*
	Male	295	40.2	39.0–41.3	38.7	17.3–66	22–59.9	
	Female	296	38.1	37.1–39.1	36.5	19–61.5	22.3–58.2	
Monocyte (MO) (%)	Combined	591	38.1	7.3–7.7	7.6	1.6–17.9	3.1–11.7	0.903
	Male	295	7.5	7.3–7.7	7.6	1.9–15.7	3.1–11.6	
	Female	296	7.5	7.2–7.7	7.6	1.6–17.9	3–11.8	
Granulocyte (GR) (%)	Combined	591	53.3	52.5–54.1	54.7	26.1–78.4	32.4–72.6	*0.024*
	Male	295	52.3	51–53.6	53.2	26.1–78.4	31.7–73.6	
	Female	296	54.3	53.2–55.4	55.5	29.8–76.0	33.5–70.5	
Platelet (PLT) (× 10^9^/L)	Combined	591	223.1	218.7–227.5	218	86–449	134–344.2	0.092
	Male	295	216.1	210.3–221.5	211	86–420	128.4–318.4	
	Female	296	230.1	223.5–236.6	226.5	93–449	145.4–351.6	

The haematological RIs established in this study were also compared to the currently used hematological RI in Asmara and associated OOR were subsequently established. The calculated values were also compared to RI established in similar studies in Tanzania, Ghana and Ethiopia (Table 3). Overall, the proportion of OOR RIs ranged from 3.5 to 46.7%. In order of decreasing magnitude, the proportion of participants who were OOR included MCV (46.7%); MCH (37.9%) and Hb (36.2%). The lowest OOR proportions were observed in RBC (3.5%); platelet count (5.4%), Hct (11.1%), RDW (11.2%), total WBC count (12.5%) and MCHC (14.9%) respectively.

**Table 3:** Comparison of the obtained RI with currently used RI and other African countries

Analyte	Sex	Newly obtained values	Ministry of Health (Eritrea)	Out of range (OOR %)	Tanzania [9]	Ghana [10]	Ethiopia [11]
Red blood cells (RBC) (× 10^12^/L)	Combined	4.07–6.02	4–6	3.5	4.01–6.12	3.39–5.83	–
	Male	4.2–6.07	–	–	4.41–6.27	3.79–5.96	3.53–6.93
	Female	4–5.7	–	–	3.84–5.59	3.09–5.30	3.45–6.25
Hemoglobin (Hgb) (g/dL)	Combined	12.6–17.7	12.5–16	36.2	11.7–17.2	9.8–16.0	–
	Male	12.6–17.8	–	–	13.7–17.7	11.3–16.4	11.5–18.0
	Female	12.5–17.6	–	–	11.1–15.7	8.8–14.4	11.0–16.7
Hematocrit (Hct) (%)	Combined	38.3–54.4	37–52	11.1	36.5–52.7	28.9–48.7	–
	Male	40.5–55	–	–	40.2–53.7	33.2–50.5	36.2–58.6
	Female	37.9–52	–	–	36.2–46.8	26.4–45.0	32.1–56.6
Mean cell volume (MCV) (fL)	Combined	85.8–100	80–94	46.7	77.6–98.1	72–97	85–100
	Male	85.7–100	–	–	76.4–98.8	70–98	–
	Female	85.5–100	–	–	77.7–97.9	73–96	–
Mean cell hemoglobin (MCH) (pg)	Combined	27.4–32.8	27–31	37.9	23.6–33.1	22.6–33.5	–
	Male	28–33	–	–	23.1–33.2	22.7–33.5	26.6–33.3
	Female	26.5–32.6	–	–	24.2–33.1	22.3–33.6	25.8–32.8
Mean cell hemoglobin concentration (MCHC) (g/dL)	Combined	30.2–33.8	32–36	14.9	30.6–34.9	30.5–36.2	–
	Male	30.4–33.7	–	–	30.6–35.1	30.6–36.0	29.5–34.4
	Female	30–33.7	–	–	30.4–34.8	30.4–36.5	28.5–34.4
Red cell distribution width (RDW) (%)	Combined	12.3–15.6	11.5–14.5	11.2	–	11.5–16.7	12–17
	Male	12.3–15.5	–	–	–	11.5–16.7	–
	Female	12.3–17	–	–	–	11.4–16.8	–
White blood cells (WBC) (× 10^9^/L)	Combined	3.4–9.0	4–9	12.5	3.0–7.9	3.4–9.2	3.2–8.8
	Male	3.7–9.3	–	–	2.8–7.9	3.5–9.2	–
	Female	3.3–8.9	–	–	3.2–8.0	3.4–9.3	–
Platelet (Plt) (× 10^9^/L)	Combined	134–344.2	150–450	5.4	150–395	89–380	128–432
	Male	128.4–318.4	–	–	147–356	88–352	–
	Female	145.4–351.6	–	–	151–425	89–403	–

Results of the comparisons of the RI obtained in this study with similar studies undertaken in Tanzania [6], Ghana [7]; and Ethiopia [8] are shown in Table 3. This study had higher RBC RI compared to the study conducted in Ghana. However, the upper limit of the combined RBC RI in the Tanzanian study was comparatively higher. Further, the RI for Hgb (12.6–17.7 g/dL) and Hct (38.3–54.4%) were higher than those obtained from Tanzania and Ghana.

## Discussion

The results obtained in this study represent haematological RI for healthy adult population in Asmara, Eritrea. The dual focus of this study was to either verify or establish new RI for specific haematological indices. The result obtained in this study underscore the fact that RI obtained in one population should not be applied to other populations.

The current study revealed that there was a gender-based difference in the RIs for specific RBC parameters including RBC count, Hgb, Hct and RBC indices like MCH and MCHC. Historically, the observed gender differences have been attributed to a range of factors including: menstruation, hormonal influences of androgen, estrogens and testosterone on erythropoiesis, and the relatively high prevalence of iron deficiency anemia in women [12, 13]. However, MCV and RDW did not exhibit a significant difference. These findings parallel findings from some studies conducted elsewhere in Africa [11, 14].

The RI of total WBC count for males and females did not show any significant variation. This is similar to findings from Ethiopia and Northern Nigeria [12, 13]. However, a study conducted in Ezurum, Turkey showed statistically significant sex-related difference for WBC counts [15].

In addition, there was no statistically significant difference in platelet count between the two sexes. These finding support similar results from studies undertaken in Ghana and Kenya [10, 16]. However, our results also indicate that the population in Asmara has lower platelet count compared to a study conducted in Pakistan (200 × 10^9^/L–390 × 10^9^/L) [17]. While the lower platelet count in this study is consistent in several African countries [16–18], its etiology is unknown.

The RI established in this study for RBC count, Hgb, Hct, MCV, MCH, and RDW differed from the Eritrean MoH comparison intervals. The upper limit for these indices tended to be higher. However, the upper RI for platelet count in the MoH comparison intervals was significantly higher, (150–450 × 10^9^/L) compared to a combined value of 134–344.2 × 10^9^/L. The OOR for RBC was 3.5% and was 46.7% for MCV. The proportion of patient misclassified by existing MCV results should be of concern.

The RBC count, Hgb and Hct RI in this study was found to be higher when compared to the study done in Kintampo Ghana [10]. This difference may be attributed to the relatively higher altitude of Asmara—Asmara (2230 m) and Kintampo (60–150 m). The reported result signifies the importance of altitude as a contributory factor to specific hematological indices. In addition, RBC indices (MCV, MCH and MCHC) were relatively higher in this study. On the contrary, the investigators in Tanzania reported lower values [9]. Interestingly, the MCV value was found to be the same as the values obtained in the Ethiopian study [11].

## Conclusion

The hematological RIs for healthy adults in Asmara established in this study differ considerably from the RI recommended by the Eritrean ministry of health and other comparison studies. There was a significant gender-based difference in the RBC count, hemoglobin, hematocrit, MCH, MCHC, differential lymphocyte and granulocyte counts. All the evaluated hematological analytes were found to be higher in males than in females except for platelet count. Further, the result obtained in this study highlight the importance of establishing population appropriate RI for an extended set of indices for the adult population in Asmara and in other African countries.

### Limitations of the study

The study had certain limitations. A fundamental limitation is that the study participants were selected on the basis of their willingness to participate in the study. Furthermore, the population in Asmara is dominated by members of the Tigrigna ethnic community. These factors might create selection bias which may limit the possibility of generalising the results obtained in this study to the entire adult population in Asmara. Further, it was also not possible to screen for all medical conditions that may have an influence on the results obtained. Irrespective of the highlighted short comings, it’s our position that the established RI may have utility for diagnostic laboratories in Asmara.

## Additional file

**Additional file 1.** Questionnaire.

## Abbreviations

ACHS: Asmara College of Health Science
CLSI: Clinical and Laboratory Standards Institute
GR: granulocyte
HBsAg: hepatitis B virus surface antigen
HcAbs: hepatitis C virus antibodies
Hct: hematocrit
MCH: mean corpuscular hemoglobin
MCHC: mean corpuscular hemoglobin concentration
Hgb: hemoglobin
IFCC: International Federation of Clinical Chemistry
k2EDTA: dipottasium ethylene diamine tetra-acetic acid
LY: lymphocyte
MCV: mean corpuscular volume
MO: monocyte
OOR: out of range
PLT: platelet
RBC: red blood cell
RI: reference interval
SPSS: Statistical Package for social services
WBC: white blood cell
