# Supplementary material for: Establishment of hematological reference intervals for healthy adults in Asmara
Source: BMC Res Notes. 2018 Jan 22;11:55. doi: 10.1186/s13104-018-3142-y (PMC5778762; doi:10.1186/s13104-018-3142-y)
Supplement: Supplementary file 1 — Additional file 1. Questionnaire. [file 13104_2018_3142_MOESM1_ESM.docx]

QUESTIONNAIRE

**A CONSENT FORM** We are intending to establish reference values of basic hematology parameters for healthy adults in Asmara for comparative and reporting purposes. So blood samples from the normal population have to be collected in sufficiently large numbers for testing In order to establish the reference ranges and your help by contributing a few cc of blood would be highly appreciated.”

Agree don't agree  SIGNATURE

**Fill the following information**

Identification code Sample ID Sex Age

Occupation Date and time collection

| 1. | Do you think you are healthy? | | YES  NO  | |
| --- | --- | --- | --- | --- |
| 2. | When was the last time you have donated blood? | | A. before 3 months B. within 3 months  C. I didn’t donate blood | |
| 3. | When have you started living in Asmara? | | A. Less than six months  B. More than six months | |
| 4. | Have you suffered from serious illness or unexpected weight loss in the last few months? | | YES  NO  | |
| 5. | Are you under a doctor’s care presently? | | YES  NO  | If yes explain? |
| 6. | Are you taking any prescribed Medication? | | YES  NO  | |
| 7. | Are there any inherited health disorders in your family? | | YES  NO  | If yes explain? |
| 8. | Do you have high Blood Pressure? | | YES  NO  | |
| 9. | Are you free from any chronic illness example diabetic, liver disease, heart disease, prolonged cough? | | YES  NO  | If No explain? |
| 10. | Whether you exposed to any modern or traditional procedure in the last six months example; Tattooing, scarification, Circumcision? | | YES  NO  | If yes explain? |
| 11. | Whether you informed to have hepatitis by a trained Medical person? | | YES  NO  | |
| 12. | Do have bleeding tendency? | | YES  NO  | |
| 13. | Did you suffer from malaria in the last 12 months? | | YES  NO  | |
| 14. | Do you experience frequent fainting attack? | | YES  NO  | |
| 15. | Do you smoke? | | YES  NO  | |
| 16. | Do you drink Alcoholic Beverages? | | YES  NO  | |
| **If you are Female;** | | | | |
| 17. | Are you menstruating presently? | YES  NO  | | |
| 18. | Are you breast feeding? | YES  NO  | | |
| 19. | Are you pregnant? | YES  NO  | | |
